# Testosterone levels and cause-specific mortality in the older French men without metabolic syndrome

**DOI:** 10.4178/epih.e2020036

**Published:** 2020-06-01

**Authors:** Nasser Laouali, Sylvie Brailly-Tabard, Catherine Helmer, Marie-Laure Ancelin, Christophe Tzourio, Alexis Elbaz, Anne Guiochon-Mantel, Marianne Canonico

**Affiliations:** 1 Paris-Saclay University, Paris-South University, UVSQ, Center for Research in Epidemiology and Population Health, INSERM, Villejuif, France; 2 Service de Génétique Moléculaire, Pharmacogénétique et Hormonologie, Hôpitaux Universitaires Paris Sud, AH-HP, CHU Bicêtre, Le Kremlin Bicêtre, France; 3 INSERM UMR_S U1185, Fac Med Paris Sud, University Paris Sud, Université Paris-Saclay, Le Kremlin Bicêtre, France; 4 University Bordeaux, INSERM, Bordeaux Population Health Research Center, UMR 1219, CIC-1401-EC, F-33000 Bordeaux, Bordeaux, France; 5 INSERM, University Montpellier, Neuropsychiatry, Epidemiological and Clinical Research, Montpellier, France; 6 University Bordeaux, INSERM, Bordeaux Population Health Research Center, UMR 1219, CHU Bordeaux, F-33000 Bordeaux, Bordeaux, France

**Keywords:** Ageing, Cancer mortality, Cardiovascular diseases mortality, Risk, Testosterone, Metabolic syndrome

## Abstract

**OBJECTIVES:**

Previous studies have reported controversial findings regarding the association of testosterone with mortality in older men. This heterogeneity might be partially explained by comorbidities and the presence of metabolic syndrome, as well as differential associations according to causes of death.

**METHODS:**

We used data from a random subsample of the Three-City study, in which hormone levels were measured in 338 men ≥65 years without metabolic syndrome who were followed-up for 12 years. Vital status was determined for all participants from different sources. We used inverse-probability-weighted Cox regression to estimate the hazard ratios (HRs) of cause-specific mortality and 95% confidence intervals (CIs).

**RESULTS:**

Over the follow-up period, 130 men died (30 from cardiovascular disease, 45 from cancer, 55 from other causes). The association of testosterone with mortality showed significant heterogeneity across causes of death (p=0.027 and p=0.022 for total and bioavailable testosterone, respectively). Higher testosterone levels were associated with increased cardiovascular mortality (HR for 1-standard deviation increase, 1.86; 95% CI, 1.28 to 2.71 and 1.50; 95% CI, 1.04 to 2.17 for total and bioavailable testosterone, respectively). By contrast, there were no significant associations of testosterone with mortality from cancer and other causes.

**CONCLUSIONS:**

Our data suggest that the association of testosterone with mortality in men without metabolic syndrome might be differential according to the cause of death. These findings may partially explain the heterogeneity across studies on the relationship between testosterone levels and mortality.

## INTRODUCTION

Findings from studies on the relationship between testosterone levels and mortality in men are highly heterogeneous, making it difficult to draw clear conclusions [1]. Identifying possible sources of heterogeneity may help better understand this relationship and to determine which groups are at higher risk. In a previous analysis, we reported that metabolic syndrome (MetS) could partially explain this heterogeneity, as there was an inverse association between testosterone and all-cause mortality in men with MetS, but not in those without MetS [2]. However, it is possible that testosterone plays a stronger role in specific causes of death, explaining the lack of association between testosterone and all-cause mortality in men without MetS. Indeed, testosterone was positively associated with cardiovascular (CV) events [3,4] but negatively with cancer [5], suggesting differential associations of testosterone with health outcomes. Nevertheless, MetS was not taken into account and no study has examined this differential risk in a metabolically “healthy” population. Specific research that enables the identification of subgroups of men at higher risk would be very important for clinical practice when considering, for example, testosterone therapy or other specific treatments. Therefore, the aim of the present study was to examine the relationship between plasma testosterone and cause-specific mortality in men without MetS.

## MATERIALS AND METHODS

The protocol of the Three-City (3C) study, a French prospective cohort study, is available elsewhere [2,6,7]. Briefly, 9,294 non-institutionalized subjects ≥ 65 years of age were selected from the electoral rolls of 3 French cities (Bordeaux, Dijon, and Montpellier) between 1999 and 2001 and interviewed 5 times (every 2 to 3 years) thereafter.

Over the 12-year follow-up, the vital status of each participant was determined based on medical records and reports from the participant’s family and physicians. A committee reviewed these data to determine immediate and underlying causes of death, according to International Classification of Diseases, 10th revision codes. Our analyses are based on the 3 main causes of death: CV, cancer, and other causes, as described elsewhere [7].

Baseline total and bioavailable testosterone were measured in a random subsample representing one-seventh of the men from the original cohort, after stratification by gender, center, and age [2]. Total testosterone was assessed by a radioimmunoassay (RIA) method with an Orion Diagnostica device (Spectria, Espoo, Finland) and a minimum detectable concentration of 0.06 nmol/L; all testosterone assays were detectable. The intra-assay and interassay coefficients of variation were 3.8% and 4.8%, respectively, for a total testosterone concentration of 10.2 nmol/L and 4.8% and 5.5%, respectively, for a total testosterone concentration of 21.3 nmol/L.

MetS was defined according to the Diabetes Federation [2,8]. Body mass index (BMI) was calculated by dividing weight (kg) by squared height (m²). Smoking status and daily alcohol consumption were considered in 3 categories (never, past, current). Education level was categorized into 3 groups (no education or primary school, secondary school, and high-school or university degree). Hypertension was defined according to the World Health Organization criteria (systolic blood pressure ≥ 140 mmHg and/or diastolic blood pressure ≥ 90 mmHg and/or blood-pressure-lowering therapy). Hypercholesterolemia was defined as total cholesterol ≥ 6.2 mmol/L and/or cholesterol-lowering therapy, and diabetes as fasting blood glucose ≥ 7 mmol/L and/or antidiabetic drugs. A personal history of CV disease at baseline included coronary heart disease (myocardial infraction, angina pectoris, coronary dilation) and stroke.

The baseline characteristics of the participants are expressed as means± standard deviation (SD), medians (interquartile range), or numbers (percentage) for categorical variables. Their distributions according to causes of death and tertiles of total testosterone were compared using the chi-square test, the Student t-test, or analysis of variance.

We used Cox proportional hazards regression models with age as the time scale to estimate hazard ratios (HRs) and 95% confidence intervals (CIs) for cause-specific mortality [9]. Participants were followed-up from baseline to the date of death from a specific cause or censoring (death from another cause or date of last confirmation that they were alive).

Model 1 was adjusted for center; model 2 was further adjusted for confounders (smoking, alcohol drinking, education level, history of CV disease, hypertension, hypercholesterolemia, diabetes, BMI). We created extra categories for covariates with missing values to keep the same number of subjects in the analyses.

In sensitivity analyses, we excluded deaths occurring during the first 2 and 4 years of follow-up.

We used a stabilized inverse-probability-weighting approach to take into account the fact that testosterone was measured in a random subcohort [10]. Weights were calculated for each individual as detailed elsewhere [2].

Statistical analyses were performed using SAS version 9.4 (SAS Institute Inc., Cary, NC, USA).

### Ethics statement

The study protocol was approved by the Ethics Committee of the University Hospital of Kremlin-Bicêtre, and all participants gave written informed consent.

## RESULTS

From the random subcohort of 495 men, we excluded 22 men who received treatments that might influence hormone levels or had a history of prostate cancer or prostatitis, 29 men without data on MetS, and 106 men with MetS. The remaining 338 men without MetS constituted the sample for our analysis.

Over 12 years of follow-up, 130 men died (38.5%): 30 from CV disease, 45 from cancer, and 55 from other causes. Compared to survivors, they were older at baseline and more likely to be current smokers and daily alcohol drinkers (for deaths from cancer) than those who were alive at the end of follow-up, independently of their age at inclusion (Table 1). As expected, men with the highest levels of testosterone were less likely to present diabetes (Supplementary Material 1).

Total and bioavailable testosterone were not significantly associated with all-cause mortality in men without MetS (HR for 1-SD increase, 1.08; 95% CI, 0.80 to 1.46 and 0.97; 95% CI, 0.73 to 1.29, respectively) but there was significant heterogeneity across causes of death (p=0.027 and p=0.022, respectively).

The associations of baseline endogenous hormone levels with cause-specific mortality are presented in Table 2. Higher total and bioavailable testosterone levels were positively associated with CV mortality (HR for 1-SD increase, 1.86; 95% CI, 1.28 to 2.71 and 1.50; 95% CI, 1.04 to 2.17, respectively). When testosterone level was categorized into tertiles, we observed a linear trend (p=0.014 and p=0.012, respectively), with a higher incidence of CV death among participants in the top tertile compared to those in the bottom tertile (HR, 2.94; 95% CI, 1.16 to 7.43 and 2.75, 95% CI, 1.15 to 6.57, respectively). There were no significant associations of total and bioavailable testosterone levels with mortality from cancer or other causes.

The results were similar after the exclusion of deaths occurring during the first 2 years and 4 years of follow-up (data not shown).

## DISCUSSION

In men without MetS, we found no significant association between testosterone and mortality overall, but we observed a differential association according to the cause of death. Independently of CV risk factors, higher levels of testosterone increased the risk of CV mortality, but were not associated with mortality from cancer and other causes.

This positive association of testosterone with CV mortality differed from some previous studies that showed an inverse association [11-13] or no association [14,15]; however, there are important differences between these studies and ours that could account for these inconsistent findings. First, other studies analyzed younger subjects than those included in the 3C, resulting in lower mortality rates. Second, these studies had shorter follow-up periods, and their findings may have been biased by reverse causation—that is, testosterone levels may have been influenced by the diseases that led to death. Reverse causation is less likely in the 3C study, given the longer follow-up and the findings of our subanalyses with the exclusion of participants who died early during the follow-up. Finally, previous studies did not take MetS status into account, while our analysis was limited to men without MetS who, by definition, have less comorbidities and are healthier compared to men overall. Conversely, our results are consistent with other studies on the risk of death from coronary heart disease [16,17] and CV events [3,4]. This is also in keeping with a meta-analysis of 27 clinical trials (n=2,994 men) showing increased CV risk in men treated with exogenous testosterone compared to placebo [18].

The mechanisms underlying the positive association between testosterone and CV mortality remain unknown. One study hypothesized that high testosterone levels may promote fluid-sodium retention in aging men, thereby contributing to the development of high blood pressure and heart failure [19]. However, in our study, adjustment for hypertension did not have a noteworthy impact. Another study suggested that the use of anabolic steroids is associated with left ventricular hypertrophy [20], thus increasing the risk of CV mortality. Although our data do not concern exogenous testosterone, we cannot exclude a similar mechanism for endogenous testosterone.

With respect to cancer mortality, we found no significant association with testosterone, in agreement with several previous studies [16,21,22]; however, other studies have shown an inverse association between testosterone and risk of cancer mortality [5,23,24]. We could not examine specific cancers due to the low number of cases and we cannot exclude the possibility that some associations might exist for specific cancers. In addition, the people who agreed to participate in the 3C study were healthier than the general population [6], and there were few current smokers at baseline. Since smoking is a very strong risk factor for CV disease and cancer, especially for aero-pharyngeal cancers, this low smoking prevalence may have led to reduced statistical power.

The major limitation of our study is that our analyses were based on a random stratified subcohort of men, rather than on the whole cohort. In order to control for sampling-related biases, we used inverse-probability-weighting [10]. In addition, testosterone measurements were carried out by RIA, rather than the state-of-the-art gas chromatography mass spectrometry method. However, validation studies showed that RIA provided accurate results in terms of relative ranking and was therefore adequate for epidemiological investigations in which population-level inferences are more of interest than subject-specific ones. Finally, the 3C cohort, like most cohort studies, is not representative of the French general population aged 65 years and over. For example, the prevalence of some CV and cancer risk factors, such as smoking status and educational level, was underestimated when compared with the general population [6]. However, we verified well-known associations and therefore we expect that this issue would attenuate, but not bias, the associations. Nevertheless, extrapolating these results to the general population must therefore be done carefully.

In conclusion, among men without MetS, high testosterone levels were found to be associated with increased CV mortality, but not cancer mortality. These findings may partially explain the heterogeneity of studies on the relationship between testosterone and mortality and help clinicians to identify groups at high-risk.

## Figures and Tables

**Table 1.** Baseline characteristics of men without MetS according to cause-specific mortality after a 12-year follow-up in the 3C cohort study

Characteristics			Alive (n=208)	CV disease mortality (n=30)	p-value^1^	p-value^2^	Cancer mortality (n=45)	p-value^1^	p-value^2^	Other cause mortality (n=55)	p-value^1^	p-value^2^
Socio-demographic												
	Age (yr)		71.8±4.3	76.3±4.2	<0.001	-	75.6±4.8	<0.01	-	77.4±5.0	<0.01	-
	Center				0.097	0.179		0.973	0.958		0.608	0.749
		Bordeaux	38 (18.3)	10 (33.3)			8 (17.8)			13 (23.6)		
		Dijon	107 (51.4)	15 (50.0)			24 (53.3)			28 (50.9)		
		Montpellier	63 (30.3)	5 (16.7)			13 (28.9)			14 (25.5)		
	Education level				0.764	0.847		0.175	0.161		0.646	0.714
		No education or primary school	49 (23.6)	8 (26.7)			15 (33.3)			14 (25.5)		
		Secondary school	54 (26.0)	9 (30.0)			14 (31.1)			17 (30.9)		
		High-school or university degree	105 (50.4)	13 (43.3)			16 (35.6)			24 (43.6)		
CV risk factors												
	BMI (kg/m^2^)^3^		25.4±2.8	26.4±2.7	0.052	0.141	25.7±2.9	0.459	0.551	25.0±3.6	0.451	0.225
	Smoking				0.043	0.044		0.019	0.024		0.910	0.823
		Never	62 (29.8)	15 (50.0)			12 (26.7)			15 (27.3)		
		Past	132 (63.5)	12 (40.0)			24 (53.3)			36 (65.4)		
		Current	14 (6.7)	3 (10.0)			9 (20.0)			4 (7.3)		
	Daily alcohol consumption				0.285	0.233		0.070	0.025		0.157	0.098
		Never	197 (94.7)	27 (90.0)			40 (88.9)			49 (89.1)		
		Past	7 (3.4)	2 (6.7)			1 (2.2)			3 (5.5)		
		Current	4 (1.9)	1 (3.3)			4 (8.9)			3 (5.5)		
	Hypertension		139 (66.8)	23 (76.7)	0.280	0.857	34 (75.6)	0.254	0.404	45 (81.8)	0.031	0.582
	Hypercholesterolemia		90 (43.3)	14 (46.7)	0.726	0.990	13 (28.9)	0.075	0.939	21 (38.2)	0.497	0.113
	Diabetes		10 (4.8)	1 (3.3)	-	-	2 (4.4)	-	-	6 (10.9)	0.112	0.560
Medical history												
	Coronary heart disease		22 (10.6)	6 (20.0)	0.134	0.617	4 (8.9)	0.718	0.387	10 (18.2)	0.125	0.119
	Stroke^4^		4 (2.0)	1 (3.5)	-	-	1 (2.2)	-	-	4 (7.7)	0.056	0.304
Biological parameters												
	Total cholesterol (mmol/L)		5.6±0.8	5.6±1.1	0.798	0.552	5.6±0.8	0.786	0.628	5.5±0.9	0.282	0.833
	LDL-C (mmol/L)^5^		3.6±0.7	3.6±1.0	0.995	0.450	3.5±0.8	0.604	0.926	3.4±0.7	0.142	0.778
	HDL-C (mmol/L)		1.5±0.3	1.4 ±0.3	0.128	0.274	1.5±0.3	0.919	0.930	1.5±0.3	0.969	0.864
	Triglycerides (mmol/L)		1.0 [0.8-1.3]	1.1 [0.9-1.4]	0.298	0.189	1.1 [0.9-1.5]	0.212	0.175	1.0 [0.8-1.3]	0.634	0.256
	Glucose (g/L)		4.9 [4.6-5.2]	4.9 [4.5-5.1]	0.520	0.611	4.9 [4.6-5.1]	0.857	0.914	4.9 [4.6-5.5]	0.153	0.055
	Total testosterone (nmol/L)		17.7±5.8	20.8±7.4	0.037	0.011	18.0±6.0	0.774	0.851	17.7±6.9	0.969	0.988
	Bioavailable testosterone (nmol/L)		10.5±3.4	11.7±3.5	0.083	0.067	9.9±3.0	0.307	0.511	10.1±3.9	0.520	0.685

Values are presented as number (%) or mean±standard deviation except for triglycerides and glucose levels, which are expressed as medians [interquartile range]. MetS, metabolic syndrome; 3C, Three-City; CV, cardiovascular; BMI, body mass index; LDL-C, low-density lipoprotein cholesterol; HDL-C, high-density lipoprotein cholesterol.

1 t-test or chi-square test.

2 Cochran-Mantel-Haenszel statistic or analysis of variance adjusted for age.

3 Missing data: n=1.

4 Missing data: n=6.

5 Missing data: n=2.

**Table 2.** Cause-specific mortality in men without MetS according to baseline testosterone levels^1^

			Events (n)	Model 1 (n=338)		Model 2 (n=338)	
				HR (95% CI)	p-value	HR (95% CI)	p-value
CV mortality							
	Total testosterone (nmol/L)						
		For 1-SD increase	30	1.61 (1.16, 2.23)	0.004	1.86 (1.28, 2.71)	0.001
		T1 (<14.95)	8	1.00 (reference)	-	1.00 (reference)	-
		T2 (14.95-19.99)	5	0.66 (0.23, 1.85)	0.423	0.64 (0.22, 1.90)	0.423
		T3 (≥20.00)	17	2.28 (1.02, 5.09)	0.045	2.94 (1.16, 7.43)	0.023
		p for linear trend			0.022		0.014
	Bioavailable testosterone (nmol/L)						
		For 1- SD increase	30	1.41 (1.02, 1.95)	0.039	1.50 (1.04, 2.17)	0.029
		T1 (<8.66)	8	1.00 (reference)	-	1.00 (reference)	-
		T2 (8.66-11.40)	4	0.55 (0.18, 1.67)	0.294	0.56 (0.17, 1.79)	0.325
		T3 (≥11.41)	18	2.41 (1.10, 5.33)	0.030	2.75 (1.15, 6.57)	0.023
		p for linear trend			0.017		0.012
Cancer mortality							
	Total testosterone (nmol/L)						
		For 1-SD increase	45	1.05 (0.79, 1.39)	0.744	1.00 (0.73, 1.35)	0.981
		T1 (<14.95)	15	1.00 (reference)	-	1.00 (reference)	-
		T2 (14.95-19.99)	16	1.20 (0.62, 2.33)	0.585	1.22 (0.61, 2.43)	0.567
		T3 (≥20.00)	14	1.00 (0.50, 2.00)	0.988	0.79 (0.37, 1.68)	0.541
		p for linear trend			0.539		0.232
	Bioavailable testosterone (nmol/L)						
		For 1-SD increase	45	0.89 (0.66, 1.18)	0.408	0.86 (0.64, 1.16)	0.318
		T1 (<8.66)	16	1.00 (reference)	-	1.00 (reference)	-
		T2 (8.66-11.40)	17	1.13 (0.60, 2.16)	0.702	1.19 (0.60, 2.32)	0.621
		T3 (≥11.41)	12	0.79 (0.39, 1.61)	0.513	0.65 (0.30, 1.40)	0.269
		p for linear trend			0.532		0.239
Other-cause mortality							
	Total testosterone (nmol/L)						
		For 1-SD increase	55	1.14 (0.87, 1.49)	0.357	1.08 (0.80, 1.46)	0.601
		T1 (<14.95)	20	1.00 (reference)	-	1.00 (reference)	-
		T2 (14.95-19.99)	18	0.77 (0.40, 1.47)	0.429	0.73 (0.37, 1.44)	0.359
		T3 (≥20.00)	17	1.04 (0.55, 1.95)	0.911	0.93 (0.46, 1.85)	0.830
		p for linear trend			0.347		0.411
	Bioavailable testosterone (nmol/L)						
		For 1-SD increase	55	1.06 (0.81, 1.39)	0.658	0.97 (0.73, 1.29)	0.831
		T1 (<8.66)	20	1.00 (reference)	-	1.00 (reference)	-
		T2 (8.66-11.40)	19	1.00 (0.53, 1.89)	0.997	0.94 (0.48, 1.83)	0.856
		T3 (≥11.41)	16	1.06 (0.56, 2.04)	0.852	0.94 (0.47, 1.88)	0.855
		p for linear trend			0.346		0.407

Model 1: adjusted for age and center; Model 2: model 1+education level, smoking status, daily alcohol consumption, personal history of coronary disease and stroke, body mass index, diabetes, hypertension, and hypercholesterolemia. MetS, metabolic syndrome; CV, cardiovascular; HR, hazard ratio; CI, confidence interval; SD, standard deviation; T, tertile.

1 HRs and 95% CIs were computed using inverse-probability-weighted Cox models.
