# Supplementary material for: Testosterone levels and cause-specific mortality in the older French men without metabolic syndrome
Source: Epidemiol Health. 2020 Jun 1;42:e2020036. doi: 10.4178/epih.e2020036 (PMC7644941; doi:10.4178/epih.e2020036)
Supplement: Supplementary Material 1. [file epih-42-e2020036-suppl.pdf]

Volume: 42, Article ID: e2020036
https://doi.org/10.4178/epih.e2020036

Spplementary Material 1. Baseline characteristics of men without MetS according to tertiles of total testosterone level in the 3C cohort study.

Characteristics	T1 (<14.95) (n=112)	T2 (14.95-19.99) (n=114)	T3 (≥20.00) (n=112)	p-trend
**Sociodemographic**				
Age (years)	73.7 (5.3)	73.4 (5.2)	73.6 (4.8)	0.969
Center				0.847
Bordeaux	24 (21.4)	23 (20.2)	22 (19.6)	
Dijon	57 (50.9)	58 (50.9)	59 (52.7)	
Montpellier	31 (27.7)	33 (28.9)	31 (27.7)	
Education level				0.417
No education or primary school	30 (26.8)	24 (21.0)	32 (28.6)	
Secondary school	30 (26.8)	28 (24.6)	36 (32.1)	
High-school or university degree	52 (46.4)	62 (54.4)	44 (39.3)	
**Cardiovascular risk factors**				
BMI (kg/m²)[a]	25.7 (3.1)	25.7 (2.6)	25.0 (3.0)	0.108
Smoking				0.309
Never	33 (29.4)	39 (34.2)	32 (28.6)	
Past	72 (64.3)	67 (58.8)	65 (58.0)	
Current	7 (6.3)	8 (7.0)	15 (13.4)	
Daily alcohol consumption				0.416
Never	105 (93.7)	105 (92.1)	103 (92.0)	
Past	4 (3.6)	6 (5.3)	3 (2.7)	
Current	3 (2.7)	3 (2.6)	6 (5.3)	
Hypertension	75 (67.0)	86 (75.4)	87 (77.7)	0.070
Hypercholesterolemia	43 (38.4)	48 (42.1)	47 (42.0)	0.587
Diabetes	9 (8.0)	8 (7.0)	2 (1.8)	0.043
**Personal history**				
Coronary heart disease	9 (8.0)	17 (14.9)	16 (14.3)	0.157
Stroke [b]	4 (3.6)	4 (3.5)	2 (1.8)	0.424
**Biologicals parameters**				
Total cholesterol (mmol/L)	5.5 (0.8)	5.7 (0.8)	5.5 (0.9)	0.965
LDL-C (mmol/L)[c]	3.6 (0.8)	3.7 (0.7)	3.5 (0.8)	0.425
HDL-C (mmol/L)	1.5 (0.3)	1.5 (0.3)	1.6 (0.3)	0.008
Triglycerides (mmol/L)	1.1 (0.8-1.4)	1.1 (0.9-1.4)	1.0 (0.8-1.3)	0.114
Glucose (g/L)	5.0 (4.6-5.3)	4.9 (4.7-5.2)	4.8 (4.5-5.1)	0.006

Data are expressed as n (%) or means (SD) except for triglycerides and glucose expressed as median (IQR).
SD= standard deviation; IQR = interquartile range; T= tertile of total testosterone level (in nmol/L); n = Numbers; BMI = body mass index; LDL-C = low-density lipoprotein cholesterol; HDL-C = high-density lipoprotein cholesterol.
p-trend based on Cochran-Armitage Test or analysis of variance.
Missing data: [a] n = 1, [b] n = 6, [c] n = 2.
